# Supplementary material for: Childhood Anemia in Mozambique: A Multilevel Mixed-Effects Analysis of 2011–2022/23 Population-Based Surveys
Source: Healthcare (Basel). 2025 Mar 14;13(6):635. doi: 10.3390/healthcare13060635 (PMC11942375; doi:10.3390/healthcare13060635)
Supplement: Supplementary file 1 [file healthcare-13-00635-s001.zip › healthcare-3402443-supplementary.pdf]

Supplementary file. Table S1. Study outcome and exposure variables.

Variables	
**Anemia in children 6-59 months**	Categorized as No and Yes.
**Anemia severity**	Categorized into the following types: not anemic (Hb ≥11.0 g/L), mild anemia (10.0 ≤ Hb ≤ 10.9 g/L), moderate anemia (7.0 ≤ Hb ≤ 9.9 g/L) and severe anemia (Hb<7.0 g/L)
**Child´s age (in months)**	Categorized into the following age groups: 6-11, 12-23, 24-35, 36-47 and 48-59 months.
**Gender of child**	Categorized as Male and Female.
**Child illness**	Categorized as No and Yes.
**Children aged 6-59 months given Vit. A supplement**	Categorized as No and Yes.
**Feeding characteristics**	
**Feeding: Group1 (cereals, roots, and tubers)**	Categorized as No and Yes.
**Feeding: Group2 (Legumes and nuts)**	Categorized as No and Yes.
**Feeding: Group3 ((Dairy products (milk, yogurt, cheese))**	Categorized as No and Yes.
**Feeding: Group4 [Flesh foods (meat, fish, fowl, liver, or other organs and eggs)**	Categorized as No and Yes.
**Feeding: Group5 (Fruits and vegetables)**	Categorized as No and Yes.
**Feeding: Group6 (Oils and fats)**	Categorized as No and Yes.
**Feeding diversity score**	Categorized into the following score groups: (0) any child who did not consume any of the food groups; (1) any child who consumed one food group; (2) any child who consumed two food groups; (3) any child who consumed three food groups; (4+) any child who consumed four or more food groups.
**Caregivers characteristics**	
**Education level**	Categorized into the following categories: No education, Primary, Secondary/Higher.
**At least 4 ANC visits**	Categorized into the following categories: less than 4 visits and 4+visits.
**Household characteristics**	
**Wealth index**	Categorized into the following categories: Poorest, Poorer, Middle, Richer and Richest.
**Sex of household head**	Categorized as Male and Female.
**Source of drinking water**	Categorized into the following categories: improved and Unimproved.
**Type of toilet facility**	Categorized into the following categories: improved and unimproved.
**All children under 5 slept under any mosquito net**	Categorized as No and Yes.

**Pollution within household**	Categorized as No and Yes.
**Community factors**	
**Residence área**	Categorized as Urban and Rural.
**Province**	Categorized as Niassa, Cabo Delgado, Nampula, Zambezia, Tete, Manica, Sofala, Inhambane, Gaza, Maputo provincia and Maputo cidade.

Supplementary file. Figure S1. Conceptual framework for child anemia determinants and consequences.

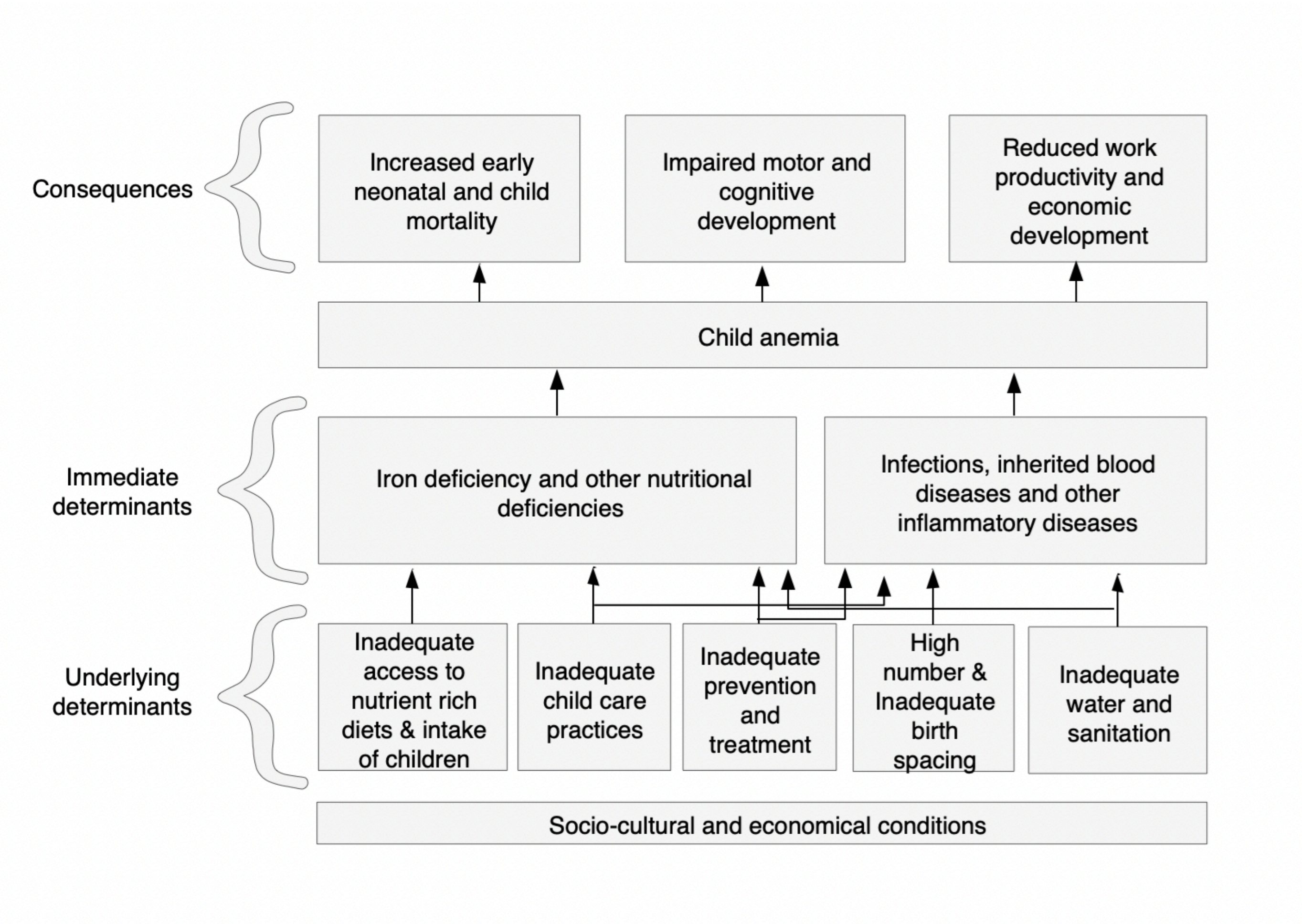

Notes: Framework adapted from:

- USAID. (2013). Conceptual frameworks for anemia. Retrieved May 27, 2024, from https://spring-nutrition.org/sites/default/files/events/multisectoral_anemia_meeting_diagrams.pdf
- Heinrichs, H., Endris, B. S., Dejene, T., Dinant, G. J., & Spigt, M. (2021). Anaemia and its determinants among young children aged 6-23 months in Ethiopia (2005-2016). *Maternal & child nutrition*, *17*(2), e13082. https://doi.org/10.1111/mc

Supplementary file. Table S2. Bivariate analysis.

**Variable**	**OR[95%CI]**
Age of the woman	0.99[0.98,0.99]*
Age of the household head	0.99[0.98,0.99]**
Birth order	1.02[0.99,1.06]
**Education level**	
No education	1.00
Primary	0.98[0.84,1.14]
Secondary/Higher	0.68[0.53,0.88]**
**At least 4 ANC visits**	
less than 4 visits	1.00
4+ visits	1.34[1.12,1.60]**
**Wealth index**	
Poorest	1.00
Poorer	0.99[0.84,1.16]
Middle	0.74[0.63,0.88]**
Richer	0.67[0.53,0.85]**
Richest	0.44[0.30,0.63]**
**Sex of household head**	
Male	1.00
Female	1.12[0.97,1.28]
**Source of drinking water**	
Improved	1.00
Unimproved	1.52[1.26,1.83]**
**Type of toilet facility**	
Improved	1.00
Unimproved	1.25[0.96,1.62]
**All children under5 slept under any mosquito net**	
No	1.00
Yes	1.01[0.93,1.11]
**Pollution within household**	
No	1.00
Yes	1.48[0.99,2.22]
**Child´s age (in months)**	
6-11	1.00
12-23	0.74[0.59,0.93]**
24-35	0.39[0.32,0.49]**
36-47	0.28[0.24,0.33]**
48-59	0.20[0.17,0.24]**
**Sex of child**	
Male	1.00
Female	0.99[0.90,1.09]
**Child illness**	
No	1.00
Yes	1.53[1.22,1.91]**
**Children aged 6-59 months given Vit. A supplement**	
No	1.00
Yes	0.84[0.69,1.03]

**Feeding characteristics**	
Group 1 (cereals, roots, and tubers)	1.33[1.18,1.49]**
Group 2 (Legumes and nuts)	1.33[1.18,1.49]**
Group 3 (Dairy products (milk, yogurt, cheese))	1.10[0.84,1.45]
Group 4 (Flesh foods (meat, fish, fowl, liver, or other organs and eggs)	1.24[1.09,1.42]**
Group5 (Fruits and vegetables)	1.24[1.13,1.35]**
Group6 (Oils and fats)	1.11[0.90,1.38]
**Residence area**	
Urban	1.00[1.00,1.00]
Rural	1.50[1.22,1.85]**

OR is unadjusted odds ratio; 95% confidence intervals (CI) in brackets; * $p < 0.05$, ** $p < 0.01$

Supplementary file. Table S3. Distribution of study variables (DHS 2011, DHS 2022-2023) (N =8143)

Variables		2011 (N=4597)<br>No. (%)	2022 (N=3546)<br>No. (%)
**Anemia in children 6-59 months**			
	No	1421 (30.9)	960 (27.1)
	Yes	3176 (69.1)	2586 (72.9)
**Child´s age (in months)**			
	6-11	594 (12.9)	393 (11.1)
	12-23	1140 (24.8)	813 (22.9)
	24-35	980 (21.3)	844 (23.8)
	36-47	985 (21.4)	745 (21)
	48-59	897 (19.5)	752 (21.2)
**Gender of child**			
	Male	2275 (49.5)	1703 (48)
	Female	2322 (50.5)	1843 (52)
**Birth order (Mean, SD)**		3.6(2.3)	3.4(2.2)
**Child illness**			
	No	3539 (77)	2916 (82.2)
	Yes	1058(23)	630 (17.8)
**Children aged 6-59 months given Vit. A supplement**			
	No	1184 (25.8)	1767 (49.8)
	Yes	3413 (74.2)	1779 (50.2)
**Feeding characteristics**			
**Feeding: Group1 (cereals, roots, and tubers)**			
	No	1720 (37.4)	2012 (56.7)
	Yes	2877 (62.6)	1534 (43.3)
**Feeding: Group2 (Legumes and nuts)**			
	No	3557 (77.4)	3115 (87.8)
	Yes	1040 (22.6)	431 (12.2)
**Feeding: Group3 (Dairy products (milk, yogurt, cheese))**			
	No	4145 (90.2)	3382 (95.4)
	Yes	452 (9.8)	164 (4.6)
**Feeding: Group4 (Flesh foods (meat, fish, fowl, liver, or other organs and eggs)**			
	No	2906 (63.2)	2895 (81.7)
	Yes	1691 (36.8)	651 (18.3)
**Feeding: Group5 (Fruits and vegetables)**			
	No	2190 (47.6)	2699 (76.1)
	Yes	2407 (52.4)	847 (23.9)
**Feeding: Group6 (Oils and fats)**			
	No	3366 (73.2)	-
	Yes	1231 (26.8)	-
**Feeding diversity score**			
	0	1467(31.9)	1895(53.4)

	1	396(8.6)	370(10.4)
	2	1175(25.6)	741(20.9)
	3	743(16.2)	399(11.3)
	4+	816(17.8)	141(4.0)
**Caregivers characteristics**			
**Education level**			
	No education	1694 (36.9)	1142 (32.2)
	Primary	2380 (51.8)	1705 (48.1)
	Secondary/Higher	523 (11.4)	700 (19.7)
**At least 4 ANC visits**			
	less than 4 visits	3728 (81.1)	3035 (85.6)
	4+ visits	869 (18.9)	511 (14.4)
**Age of the woman (Mean, SD)**		28.9(7.2)	28.4(7.5)
**Household characteristics**			
**Wealth index**			
	Poorest	1100 (23.9)	949 (26.8)
	Poorer	1046 (22.7)	815 (23.0)
	Middle	871 (18.9)	699 (19.7)
	Richer	924 (20.1)	650 (18.3)
	Richest	656 (14.3)	433 (12.2)
**Sex of household head**			
	Male	3271 (71.1)	2682 (75.6)
	Female	1326 (28.9)	864 (24.4)
**Age of the household head (Mean, SD)**		36.7(11.7)	37.5(12.3)
**Source of drinking water**			
	Improved	1622 (35.3)	1983 (55.9)
	Unimproved	2975 (64.7)	1563 (44.1)
**Type of toilet facility**			
	Improved	1062 (23.1)	910 (25.7)
	Unimproved	3535 (76.9)	2636 (74.3)
**All children under 5 slept under any mosquito net**			
	No	2964 (64.5)	2159 (60.9)
	Yes	1633 (35.5)	1387 (39.1)
**Pollution within household**			
	No	80 (1.7)	89 (2.5)
	Yes	4517 (98.3)	3458 (97.5)
**Community factors**			
**Residence area**			
	Urban	1210 (26.3)	979 (27.6)
	Rural	3387 (73.7)	2567 (72.4)
**Province**			
	Niassa	274 (6.0)	309 (8.7)
	Cabo Delgado	382 (8.3)	234 (6.6)
	Nampula	708 (15.4)	975 (27.5)
	Zambezia	1011 (22.0)	613 (17.3)
	Tete	588 (12.8)	382 (10.8)
	Manica	355 (7.7)	276 (7.8)
	Sofala	441 (9.6)	253 (7.1)

	Inhambane	221 (4.8)	120 (3.4)
	Gaza	223 (4.8)	129 (3.6)
	Maputo provincia	240 (5.2)	184 (5.2)
	Maputo cidade	156 (3.4)	72 (2.0)
**Notes:**	SD: standard deviation		
